# Outcomes of patient and public involvement in the development of the Cognitive Decline after Brain Radiosurgery (CoDe B-Rad) study: refining the research question and methodology

**DOI:** 10.1136/bmjopen-2024-094788

**Published:** 2025-06-26

**Authors:** Anna Bangiri, Adele Horobin, Julia Baker, Stefan Pszczolkowski, Stefanie Thust, Paul S Morgan

**Affiliations:** 1 Radiotherapy North, Nottingham University Hospitals NHS Trust - City Campus, Nottingham, UK; 2 Radiological and Imaging Sciences, University of Nottingham School of Medicine, Nottingham, UK; 3 University of Nottingham, Nottingham, UK; 4 Patient and Public Involvement Contributor, Doncaster, UK; 5 Radiological Sciences, Mental Health and Clinical Neuroscience, University of Nottingham School of Medicine, Nottingham, UK; 6 Division of Clinical Neuroscience, University of Nottingham School of Medicine, Nottingham, UK; 7 Non-Ionising Imaging Physics, Nottingham University Hospitals NHS Trust, Nottingham, UK

**Keywords:** Cognition, ONCOLOGY, Quality of Life, Radiosurgery, Patient Participation, Patient Reported Outcome Measures

## Abstract

**Objectives:**

Patient and public involvement (PPI) was sought in the development of the protocol for the Cognitive Decline after Brain Radiosurgery (CoDe B-Rad) study, which aims to identify potential side effects of stereotactic radiosurgery (SRS). PPI served to refine the research question and methodology.

**Design:**

PPI.

**Setting:**

PPI conducted online with people based in the UK. The CoDe B-Rad study is running in regional National Health Service tertiary care in the UK and is currently nearing recruitment completion.

**Participants:**

Patients and carers with lived experiences of brain radiotherapy. Contributors were identified through national charities.

**Procedures:**

Initial focus groups were planned, but participation proved challenging. Instead, online questionnaires, one-to-one discussions and participation in support groups were completed.

**Results:**

All contributors experienced changes to their cognition and/or quality of life (QoL) after radiotherapy. Quantifying the side effects of SRS and minimising them were identified as a research gap. Discussion group participation proved challenging. PPI plans were altered to accommodate the physical and mental needs of contributors. It was decided to combine the Montreal Cognitive Assessment along with European Organisation for Research and Treatment in Cancer QLQ-C30 and BN20 to capture cognitive status and QoL of patients with brain metastases and meningiomas after SRS. Patients/carers recommended for sessions to be restricted to 30 min and testing to be offered face-to-face, online, in hospital or at patients’ homes. Coproduction was not achievable with our patient population but that did not diminish the input of contributors nor the impact it had on designing the study protocol.

**Conclusions:**

In cancer research, diligent considerations are required to ensure the suitability of involvement methods for this vulnerable population. Flexibility and adaptability of draft PPI plans are essential to achieve meaningful contributions. The protocol of the ongoing CoDe B-Rad study was positively shaped by people with lived experiences of brain radiotherapy.

**Trial registration number:**

NCT06466720 (CoDe B-Rad study).

**STRENGTHS AND LIMITATIONS OF THIS STUDY**

- All input from patients and carers, including comments and answers, was used to shape the study protocol.
- Good relationships with contributors were built, which led to honest and open conversations.
- This patient and public involvement (PPI) strategy was applied in a flexible and adaptable way and in response to the challenges faced when working with a frail and vulnerable patient population; feedback on the study progression was given and informative webinars for patients were provided by way of thanks.
- No patients/carers or people with extensive PPI experience were included in the development and delivery of the PPI strategy.
- The total number of contributors was lower than expected, and consequently, a diverse range of voices was not achieved.

## Introduction

 Patient and public involvement (PPI) in research is defined as “research carried out ‘with’ or ‘by’ members of the public rather than ‘to’, ‘about’ or ‘for’ them” by the UK National Institute for Health Research.^1^ This definition is echoed in the US and Canadian research infrastructures and national frameworks, which include the Patient-Centered Outcomes Research Institute and the Patient Engagement Framework, respectively.^2^,^4^

Building on Arnstein’s earlier work,^5^ Indigo Daya developed the ‘participation ladder’, which provided a framework to more reproducibly categorise PPI activities.^6^ The ladder progresses from exclusion, which lies at the bottom, to information/education to engagement/consultation, followed by co-design/co-production and finally user owned/led activity, which forms the top of the ladder.^5 6^ Molony-Oates adapted this participation ladder and applied it to patient and public involvement in research in the UK.^7^

Cancer patients can be an extremely vulnerable population. They can remain seriously ill over time or move into survivorship, frequently with long-term debilitating side effects^8 9^ and the psychological burden of a relapse risk. Care should be taken to meet the specific needs and preferences of these patients and their carers when planning PPI activities.^10^

Hubbard *et al* showed that involving people in research who have been affected by cancer, directly or indirectly, has enriched research design and improved recruitment as well as response rates. Specifically, this involvement allowed researchers to interact with groups that would otherwise be under-represented.^11^ Bergin *et al* in their systematic review of PPI in cancer research observed that the positive impacts of PPI led to improved study designs, more sustained recruitment and a higher chance of clinical implementation of research findings among other advantages.^12 13^

In this paper, we document our learning from seeking to involve brain tumour patients and their carers in developing new research ideas and a study protocol for the Cognitive Decline after Brain Radiosurgery (CoDe B-Rad) pilot study. Brain tumour patients can be particularly unwell and, in some cases, may be under palliative care. We share the impact that involving such individuals and their families had on the research plans, and our reflections on how best to involve this population in the future. The aim of this PPI was to (1) identify the perceived side effects of radiotherapy treatment, (2) understand their impact on patients, and (3) design research around what people with lived experience were sharing with us. We share our learning from this involvement.

## Methods

We followed recent guidance by Staniszewska *et al* on how to report PPI in research.^14^ The Guidance for Reporting Involvement of Patients and the Public 2 (GRIPP2) short form was adopted to report patient and carer involvement in the development of the research protocol for the CoDe B-Rad study.^14^

The first step of protocol development focused on understanding the most common and the most challenging symptoms, which brain radiotherapy patients experience and what they would like to see addressed by research. This aimed to build a study around patient priorities. The second step focused on using these patient-identified priorities to help us define the most appropriate patient population to target. The third step focused on inviting members and carers of our target patient population to help us refine the study design and patient-facing material. Throughout, a flexible approach was maintained to maximise communication and fit around the needs of the patients and carers involved.

One researcher (AB) formulated the original PPI plans, questions for the open discussions, the online questionnaires and the one-to-one discussions without any prior PPI input. The work was conducted in late 2023.

### Understanding the impact of brain radiotherapy treatment on patients

#### Open discussions with patients and carers

Through contact with relevant charities, the plan was to hold discussion groups for patients and carers or join discussions of existing patient support groups. Three charities were contacted: Maggie’s at Nottingham (www.maggies.org), The Brain Tumour Charity (www.thebraintumourcharity.org) and brainstrust (https://brainstrust.org.uk).

Maggie’s invited the research team to join an East Midlands-wide support group meeting held online. AB attended the meeting with the prior consent of the people attending. Five patients and two carers attended, and AB introduced herself and listened to their discussions. AB asked those present to share their experiences of radiotherapy, if they wished to do so. AB also asked for their views on the impact of radiotherapy, what side effects, if any, they had experienced and which of those persisted. AB enquired about their needs and what they would like future research to focus on. A good rapport was built with those present, and AB offered to have more detailed one-to-one discussions following the meeting with anyone who wished to do so.

Two members from the Maggie’s support group, both patients, agreed to talk further. On a one-to-one basis, the same topics as discussed before were revisited, with each member having more time and space to share their personal experiences.

The Brain Tumour Charity (www.thebraintumourcharity.org) and brainstrust (https://brainstrust.org.uk) could not link the research team with any support groups. Instead, the team arranged to hold discussion group meetings, and the charities kindly advertised the opportunity to join these meetings to their members.

Brainstrust and The Brain Tumour Charity acted as brokers with patients and carers to gather sufficient interest for the discussion groups to be held. However, closer to the date, both charities reported that the majority of those who had shown interest could not attend and so neither of the discussion groups could go ahead. Instead, the team offered one-to-one discussions for anyone who could still contribute.

One discussion was held with a patient and a second one with an unrelated carer. AB asked both to share their experiences of radiotherapy treatment, the symptoms experienced and which, if any, of those had persisted. Finally, AB asked both contributors what they thought was the impact of radiotherapy on them/their loved ones. Participants were also asked what they would like future research to focus on.

#### Questionnaires for patients and carers

There was a need to gather the views of a larger number of people while keeping the burden of involvement as low as possible. To adapt, a short questionnaire was created, which the charities agreed to distribute to their members. Respondents could reply anonymously, and in their own time. The same questions were posed in the questionnaire, as in the one-to-one discussions and the patient support group, and the wording was kept as brief as possible. The questionnaire was accessible online (online supplemental material 1). Seven people responded, each completing the answers to all questions.

### Defining the patient population

Equipped with a better understanding of the impact of brain radiotherapy treatment on patients, including the symptoms the research should focus on, we held informal discussions with clinical stakeholders (one neuro-radiologist and two clinical oncologists) to understand which patient populations we should involve. The discussions were centred around the following:

- Clinical characteristics of patient populations.
- The care each population receives.
- Their experiences of the difficulties that patients are faced with.
- The research aim to map radiotherapy doses against symptoms.

This helped in defining which patient groups to build the study around.

### Refining the study design around the needs of the patient population

Once the patient population had been defined, we sought the views specifically of patients and carers within this. We were particularly conscious of the vulnerability of these patients as research participants. To maximise dissemination, we approached patients and carers using an online questionnaire (online supplemental material 2). The brainstrust and Brain Tumour Charity charities distributed this to their members through their newsletter. Via this questionnaire, we sought thw following:

- Confirmation from patients that the planned research items indeed corresponded to the most common and/or challenging symptoms for them, as reassurance that the research team pursued the correct priorities.
- Further in-depth insights to inform the most appropriate research outcomes and measures the research team should implement in the study design to minimise the burden of participation.

The questionnaire included queries about side effects experienced from treatment and their impact on the daily lives of patients/carers, the length and frequency of testing that would be acceptable, and whether or not quality of life (QoL) measures should be collected. Furthermore, opinions were sought on where the tests should take place (the hospital, online, the patient’s home or all of them), and the format in which the tests should be delivered (paper or electronic). A total of three people responded to this questionnaire.

Additionally, one patient from the target patient population agreed to have a one-to-one discussion with AB. The discussion topics were closely aligned to those from the questionnaire.

Following patient and carer input, we discussed and agreed upon a study protocol. The team’s decisions on the most appropriate testing instruments and testing intervals were guided by the questionnaire responses and notes from the one-to-one discussion.

We provided feedback to all contributors on the difference their input made to the study design and ongoing progress, both directly and through the charities.

#### Refining patient-facing material

After the study protocol was fully drafted, prior to submission for study ethical approvals, the Participant Information Leaflets (PIL) and the lay summary were reviewed by three members of the public. They were identified through the brainstrust’s Patient Research Involvement Movement.^15^ Their comments were incorporated into the revised versions of the documents.

### Patient and public involvement

Patients and carers were involved during protocol development for the CoDe B-Rad study. Contributors, through their lived experiences, helped refine the research question. They also advised on the burden of the intervention, the overall testing length and interval. When the study results become available, contributors will be consulted on the best way to disseminate the information to the wider community.

## Results

### Understanding the impact of brain radiotherapy treatment on patients

#### Open discussions with patients and carers

A total of five patients and two carers attended Maggie’s East Midlands online support group meeting in late 2023. They raised a number of side effects and their impacts from radiotherapy treatment during discussions and suggested future topics for research (table 1).

**Table 1:** Summary of open discussions with patients and carers about side effects from brain radiotherapy and their suggestions for future research

Side effects/symptoms	FatigueSight lossDifficulties with: Memory Word finding Multitasking Visuospatial perception Loss of libido and erectile dysfunctionChanges in tasteLoss of ‘intelligence’Loss of function on one side of the body
Which symptoms were most impactful (physically and/or psychologically)	Loss of ‘intelligence’Fatigue (severe and/or debilitating)Difficulties with memoryChange in taste (radical change in diet)Loss of libido (severe psychological impact)
Future focus of research	Reducing the cognitive side effects of radiotherapyLearning more about side effects and their psychological impact

Patients who had built careers around mentally demanding work felt the ‘loss of intelligence’, and memory issues, profoundly. They perceived their cognitive abilities to be intertwined with their sense of self. Losing these abilities and with them their careers meant losing their sense of identity, with deep psychological impacts. They advised that it was important to capture the mental health status of patients and recommended that study participants also complete quality of life questionnaires (QLQs).

AB asked the group whether any cognitive assessment had been offered during treatment or as part of the aftercare. Contributors confirmed they had not, apart from one patient who reported receiving an assessment, after their surgery. One patient recommended including cognitive assessments in routine care and others agreed. However, two contributors thought it might worsen the mental health of patients who are prone to depression.

One-to-one discussions were held with two people from the support group and two (one patient and one carer) following the discussion group meeting cancellations. Key points they raised during discussions are summarised in table 2.

**Table 2:** Summary of one-to-one discussions with patients and carers about side effects from brain radiotherapy and their suggestions for future research

Side effects/symptoms	FatigueDifficulties with: Memory Word finding Multitasking Visuospatial perception Personality changesHair lossLoss of libido
Which symptoms were most impactful (physically and/or psychologically)	Hair loss (psychological impact)Loss of libido (psychological impact that affected relationship with partner)Personality changes (impacted on relationships with family/friends)Issues with multitaskingIssues with word finding
Future focus of research	Cognitive testingLearn as much as possible about the side effects from radiotherapy

All contributors agreed that having cognitive testing as part of routine care would have given them a baseline to compare against future change and assisted them in seeking help. The carer also pointed out that the patients might have an erroneous sense of how well their brain is functioning and “might not remember, what they don’t remember”.

All agreed that it is important to find out as much as possible, as early as possible, about the side effects of the radiotherapy treatment. This would help patients make informed decisions and identify and seek help for symptoms earlier.

#### Questionnaires for patients and carers

Seven people answered the questionnaire, with one identifying themselves as a carer. All respondents had, or cared for someone with, primary brain tumours and had received radiotherapy. In addition, four had surgery as part of their treatment and six had chemotherapy. Three out of seven still had ongoing treatment, whereas the remainder had finished with all treatment as a minimum 6 months prior. Responses are summarised in table 3.

**Table 3:** Summary of the replies from the questionnaire for patients and carers

Side effects/symptoms	Balance issues/dizzinessFatigueHair loss and changes to hair growthLoss of strength and one-sided weaknessDifficulties with: Multitasking Memory Word finding/communication Concentration Bladder control Apathy, confusion and frustrationErectile dysfunction	
Symptoms that improved with time	Hair loss (one patient)Fatigue (one patient)All physical symptoms (for two patients)All symptoms improved (for one patient)Balance issues (one patient)General physical health (one patient)Mobility and clarity (one patient)	
Symptoms that did not improve or got worse with time	No improvement in any symptoms (one patient)Issues with memoryIssues with communicationAll the cognitive symptoms (for one patient)	
Which symptoms impacted on patient’s daily lives (physically and psychologically)	FatigueLoss of independenceNot being able to workHair lossIssues with speech/communication	
Impact of side effects on daily life	For five out of seven patients, the side effects were impacting their lives daily (4 or 5 on the scale)For 2 patients, the impact was small (2 on the scale)	
	Likert scale	Number responses
	1 (No impact)	0
	2	2
	3	0
	4	2
	5 (Daily impact)	3
Future focus of research	Improving and reducing side effects of radiotherapyBetter information for patients on potential side effects and potential benefits so they can make informed decisionsImproving quality of lifeMental confusionEffect of treatment on eyesightWhy treatment affects people in different ways	

From discussions and the questionnaire, we found all contributors that had undergone radiotherapy reported side effects, many of which impacted on their daily lives. Many reported some form of cognitive impairment or decline and effects on their mental health. Side effects were both short and long term. Interest in research centred around understanding, explaining and addressing side effects. This directed our study focus towards identifying areas in the brain that are linked to cognitive decline or other physical and mental health symptoms (such as fatigue, hair loss or social functioning) after brain radiotherapy.

### Defining the patient population

After discussions with clinical stakeholders and identifying available patient numbers within each population, the research team decided to focus the study on patients with brain metastases or meningiomas who undergo stereotactic radiosurgery (SRS).

Brain metastases and meningiomas are the two most common brain tumours. Brain metastases occur when cells from tumours originating outside of the brain travel through the bloodstream and deposit themselves in the brain.^16^ They are the most common brain tumour, with 20–40% of all cancer patients being affected.^17^ Kurian *et al* estimated that approximately 16 000 patients live with brain metastases in the UK.^18^

Meningioma is the most common primary tumour, accounting for 27% of all brain tumours diagnosed in the UK between 1995 and 2017.^19^ This arises from the meningeal coverings (dura) with the potential to compress and occasionally invade brain tissue. Radiotherapy is commonly reserved for cases of malignant and recurrent benign meningiomas.^19^

Although brain metastases and meningiomas markedly differ in pathobiology, both are tumours that do not extensively infiltrate distant brain tissues in the way that certain brain cancers such as diffuse gliomas do.^20^ Therefore, localisation and attribution of cognitive impairment due to treatment effects are feasible. Additionally, both tumour types can be treated effectively with SRS; however, SRS side effects, especially on cognition, have not been studied in detail due to the presumption that focal radiotherapy spares healthy tissue.^21 22^ To distinguish between long- and short-term side effects and different life expectancies of patients, two cohorts will be defined: a retrospective cohort, consisting solely of meningioma patients, and a prospective cohort, consisting of meningioma and brain metastases patients.

### Refining the study design around the needs of the patient population

Three people responded to the questionnaire specific to the brain metastases patient population (table 4). A one-to-one discussion was held with a brain metastases patient. They reported on their symptoms, and the summary of all replies is shown in table 4.

**Table 4:** Results from the questionnaire for patients with brain metastases only

Symptoms	One-sided weakness/poor mobility Fatigue Hair loss Loss of hearing Headaches Slurred speech Facial spasms
Which symptoms impacted on patient’s daily lives (physically and psychologically)	Cognitive symptomsFatigue
Impact of side effects on daily lives (scale of 1 to 5, where 1 is none and 5 is daily impact)	Cognitive issues had a large impact on daily life (5 on the scale)Mental health was also impacted upon (a scale was not used for this); 2 out of 3 reported an impact
Tests, format and testing time	Quality of life measures should be includedFormat: option available for online or paper-based testing30 min maximum (2 out of 3 contributors)Up to 1 hour (one contributor)
Testing interval	*Consensus not reached*Baseline and 1 month post treatmentBaseline and 3-monthly intervalsBaseline and 6-monthly intervals
Location of testing	*Consensus not reached*Hospital (around other appointments)Patient’s homeA combination of the above

Contributors reported that the cognitive side effects, along with fatigue, were the most impactful on their daily lives. They also reported an impact on their mental health and agreed that QLQs need to be included in the testing. Consensus on the time interval was not reached, while two out of three contributors suggested a maximum of 30 min for all testing to take place. Additionally, consensus was not reached for the location of the testing, and therefore, the research team chose to offer all options, as well as include online testing.

#### Deciding on testing instruments

The main issues identified by the contributors were centred around cognition and its impact on their daily lives. In radiotherapy, it is common to identify, outline and protect organs at risk, such as the eyes, optic nerves, spinal cord and others in order to minimise dose exposure during treatment. This leads to fewer side effects and complications for patients. However, no thought is given towards minimising cognitive side effects, such as those reported by our contributors. As such, the research team decided to focus the project on measuring and mapping cognitive decline and QoL to different areas of the brain.

The aim is to identify areas of the brain that play a role in cognition, or other measures of QoL, and treating these areas as ‘organs at risk’. Subsequently, the aim is to minimise radiotherapy dose exposure to those areas of the brain, without compromising overall dose and therapeutic efficacy. Table 5 shows the cognitive symptoms identified by contributors mapped against cognitive domains. These align well with the symptoms identified by the National Institute of Health and Care Excellence in the UK and those reported in the literature.^23^,^29^ A cognitive test that includes all these domains will be used, to ensure that the majority of, if not all, patient-reported symptoms are covered. The length of the test will be considered to ensure alignment with the suggestions from contributors.

**Table 5:** Mapping of the cognitive symptoms against the relevant cognitive domains

Symptom	Cognitive domain
Memory problems	MemoryExecutive function
Word finding issues	FluencyLanguage
Issues with spatial awareness	Visuospatial function
Inability to multitask	Executive functionLanguage

After discussions with neurologists and neuro-psychologists, the Montreal Cognitive Assessment (MoCA) will be used in the study.^30^ The MoCA incorporates testing of all the cognitive domains identified as important by the patients and carers. It takes between 10 and 15 min to complete, allowing for the QLQs to be completed within 30 min.^30 31^ Finally, it has been previously tested on patients with brain metastases, as well as primary tumours.^31^,^33^ Based on the contributions of patients and carers, QLQs will also be used to assess the QoL of the participants, their mental health and the impact that some of the symptoms are having on the participants’ daily lives. The QLQs selected were developed by the European Organisation for Research and Treatment in Cancer and their use is specific for patients with brain tumours. They are QLQ-C30 and BN20.^34^,^36^ These questionnaires are appropriate for both brain metastases and meningioma patients. They can both be completed within 15 min.

#### Deciding on testing intervals

Patients with brain metastases have a median life expectancy between 6 and 12 months.^37^ In the UK, patients who have been treated with SRS are not allowed to drive.^23^ They have 3-monthly follow-up appointments for MRI scans to review the effects of initial SRS and monitor for new metastases.

Considering these patients are in the palliative stage of their disease, and their transport options are restricted, the protocol was designed to interfere as little as possible with their daily lives. In lieu of any consensus from the PPI members we consulted, the interval for cognitive testing and for completing the QoL questionnaires was set at baseline and 3-monthly intervals post treatment, to coincide with routine clinical care. The option of carrying out the testing online or face to face would be offered, as well as the possibility of completing the tests either in hospital (around other scheduled appointments for standard of care) or at home. The MoCA can be delivered with a 3-monthly interval without a learning effect.^38^

The majority of patients with meningiomas have longer life expectancies. According to Cancer Research UK, their survival ranges between approximately 70% and 40%, at 10 years, for low-grade and high-grade tumours, respectively.^39^ The research team decided that for consistency, all patients would undergo the same methods of cognitive tests and QLQs and be tested at the same time intervals as the brain metastases patients.

With regard to testing, the following options will be given to patients, as per the suggestions of patients and carers. The tests will be available in paper and electronic form. Both the cognitive test and the QLQs are available in more than 100 languages, to facilitate maximum inclusion.

All patient and carer contributions along with the respective changes in the protocol are summarised in table 6.

**Table 6:** The entirety of the patient and carer contributions for this project, including how they were made, and what action the research team took to incorporate them in the study

Patients and carers said	How they contributed	How we responded
Focus on side effects and minimising them	Group discussionOne-to-one discussionsQuestionnaires	Focused the study on cognitive decline and physical and mental symptomsMeasuring and mapping symptoms across the brain so that we can reduce side effects in future patients
Quality of life can be affected severely	Group discussionOne-to-one discussionsQuestionnaires	Included two quality of life questionnairesFunctional and emotional scales will also be mapped across the brain to identify areas of interest
No more than 30 min of testing	Questionnaire	Identified cognitive test and QLQs can be completed within 30 min
Offer flexibility	Questionnaire	Tests will be offered online or on paper, in hospital or at home
Mental Health Burden	Group discussion	Added information in the Patient Information Leaflet and created a ‘mental health support pack’
Minimal burden	Group discussionQuestionnaire	All additional testing to coincide with clinical appointments and follow-up

The changes made to the protocol were fed back to all contributors in the forms of newsletters, which were distributed by each charity. The contributors were also informed about the progress of the research project, when the ethics application was submitted, and approval was granted.

### Refining patient-facing material

Contributors suggested removing the images from the original PIL, as they found them distracting. The formatting of certain parts of the documents was amended, to make them more accessible and clearer. Left alignment was used and shorter paragraphs with more spaces in between. They pointed out that using words like ‘cohort’ and ‘lesion’ could be confusing for patients. The words were changed to ‘groups’ and ‘tumour’, respectively.

## Discussion

Hubbard *et al*, in their review in 2008, found that it was mostly breast cancer patients that were involved in cancer research.^11^ However, the landscape is changing with Bergin *et al* reporting on studies of more than 10 different types of cancers with thousands of participants, supported by 2 to 60 PPI members,^13^ and this is echoed in the systematic review by Hoffmann Pii *et al*.^10^ In this study, we involved patients and carers with primary and secondary brain cancers.

### Impact of PPI on study design

Patients and their carers directed the research team towards focusing on measuring cognitive side effects and QoL using short tests of up to 30 min. This contrasted with the research team’s original plans that focused solely on cognition using testing instruments which could take up to an hour to complete.

Patients and carers identified mental health as an important and easy to overlook aspect of their lives that had been impacted by treatment. This was echoed by all those that answered the questionnaires. Consequently, two QLQs have been newly added onto the research protocol. The questionnaires selected have been specifically designed for use with brain tumour patients and can also be completed within 15 min.

Another key contribution of the PPI was in highlighting the potential impact of completing cognitive tests and QLQs on the mental health of participants. In response, a ‘mental health support pack’ was collated with details of mental health support available. This will be offered to any participants who become upset. Finally, in their review of the lay summary and the PIL, a clearer representation of the information to potential participants and corrections to use more accessible language has been achieved.

The resulting CoDe B-Rad study aims to identify areas in the brain that are linked to cognitive decline and physical and mental health symptoms after brain SRS. Neurocognitive testing is not routinely undertaken as part of the standard of care for patients with brain tumours in the National Health Service (NHS) in the UK. Testing only takes place if the clinician or the patient themselves voice concerns about cognitive function. Consequently, evidence is lacking in the literature^21^ and in clinical practice about the direct effects of radiosurgery on cognitive function. CoDe B-Rad will measure the potential side effects of radiosurgery and map them spatially across the entire brain using voxel-based lesion symptom mapping.^40^

### Challenges

Getting enough patients to participate in a discussion group proved challenging. Two discussion groups were organised, with approximately 3 weeks’ notice given of the date and time, but could not go ahead due to late cancellations. One reason could be, as patients explained during the support group sessions, that physical and mental health can vary greatly from 1 day to the next. The symptoms are inconsistent and unpredictable in their severity. Had there been patient and carer input into developing the initial plans, this issue might have been anticipated and accommodated. This is a limitation of the study, with regard to developing the initial PPI plans.

The low number of contributors is another limitation, and due to that, diversity in the range of voices was not achieved. Such barriers when aiming to partner with frail or seriously ill patients have been detailed by Ludwig *et al*,^41^ Bates *et al*^42^ and Caldon *et al*.^43^ Stephens *et al* struggled to recruit patients to sit on the steering group of their James Lind Alliance Priority Setting Partnership for mesothelioma.^44^ Perkins *et al* highlighted that for terminally ill patients, answering a questionnaire is a more feasible activity than attending a discussion group.^45^ Providing ongoing feedback can also prove to be challenging, when working with patients with short life expectancy, as they may have passed away by the time the study commences or is completed.^46^

For the brain metastases population specifically, the life expectancy is limited, and patients can deteriorate rapidly. As such, the formation of a discussion group consisting exclusively of affected patients can be difficult to achieve. Moreover, it is unlikely that the majority of patients would be able to participate in a patient advisory group or in the research team for the duration of a study. Consequently, coproduction was not achieved. Regardless, the lived experiences of contributors had a significant impact on the study design of the CoDe B-Rad study.

### Reflections

The experience that the team had with patients and carers was extremely positive and helped identify issues that could not have been foreseen by the researchers alone. They provided invaluable insight with regard to treatment effects and how their impact can differ in different populations (short- vs long-term survivors). In addition, the contributors identified areas of interest, such as QoL or the potential mental health impact of underperforming during a test, that the researchers did not have in their initial scope.

The contributions of patient and carers to the development and refining of the protocol for the CoDe B-Rad study have been multiple. Contributors were eager to share their experiences and help in any way possible. Their motivations were altruistic in that by advising researchers, they would help benefit future patients. In addition, as these individuals were already participating in support groups, they were used to sharing experiences and were happy to welcome healthcare professionals that wanted to listen to them. As a researcher, it was a grounding and positive experience to meet with patients and carers, listen to their views and read their feedback in questionnaires.

Attending the patient support group provided a deeper insight into the views of contributors. The contributors felt safe in a familiar environment and comfortable in discussing even ‘sensitive’ topics such as loss of libido and erectile dysfunction. Being mindful of the context within which researchers and members of the public interact is crucial to checking one’s own power and developing inclusive practice.^47^

The relationship dynamic between researchers and contributors is an interesting topic.^11^ It is often easier for the researcher to arrange group and one-to-one meetings in the researcher’s area of study or work. However, not all members of the public feel completely at ease in an unfamiliar environment, and this could impact negatively on building a productive and mutually beneficial researcher-contributor relationship. Arranging meetings in neutral environments can help build relationships and allow contributors to present their view more freely.^48^ Moreover, researchers joining patients and carers in their environments, such as an established support group of a community, can attenuate, or even reverse, usual power dynamics.^47^ Meeting patients and carers in their comfort zone rather than in the researcher’s helps break down institutional barriers, address any power imbalance and build trust.

The input from contributors was invaluable and all the changes that they suggested were implemented in the study protocol. When aiming to include patient and public partners in research, any potential physical and mental limitations within the population need to be considered. PPI plans might have to be adapted to allow, increase or sustain involvement from contributors, and this might occur at any point within a research project. One size does not fit all, and research teams, as well as funders, should be aware of this when preparing or assessing PPI plans, respectively. Adapting to their needs is the best strategy for successful PPI.

The research team offered to reimburse participants for their contributions. However, the majority declined. A lunch could not be offered as a thank you, since contributors were living across the East Midlands and in some cases even further away. Patients with brain tumours are only allowed to drive under very specific circumstances and therefore are dependent on family and friends or public transport for mobility. Consequently, after discussions with the facilitator of the Maggie’s brainwaves support group, it was decided that a payment would be made to support a Tai-Chi session in the Nottingham centre. In this way, anybody attending the session could benefit. Additionally, two webinars for patients, carers and the public were delivered by AB through brainstrust and Maggies’. AB gave an insight into the details of the radiotherapy journey and what happens in the background, in preparation for each patient treatment. These initiatives were well received and demonstrate that there are other ways to value, recognise and reward patients and carers for their contributions.

### Conclusion

We have reported the PPI activities which positively shaped the protocol of the CoDe B-Rad study. PPI contributors highlighted physical and mental health symptoms to consider, advised on serial testing duration and intervals and identified ways of tailoring the planned research to the vulnerability of the study population. By this, the testing time was restricted, at-home testing was offered and the research coincides with clinical follow-up.

The researchers found the experience of involving patients and carers in designing the study extremely valuable. Each contributor provided a new insight into the patient experience. Consideration should be given when working with physically and mentally unwell patients that activities might not go as planned and last-minute cancellations might require adaptable strategies for PPI.

## Supplementary material

10.1136/bmjopen-2024-094788 online supplemental file 1

10.1136/bmjopen-2024-094788 online supplemental file 2

## Data Availability

Data sharing not applicable as no datasets generated and/or analysed for this study. No data are available.
