# Supplementary material for: Outcomes of patient and public involvement in the development of the Cognitive Decline after Brain Radiosurgery (CoDe B-Rad) study: refining the research question and methodology
Source: BMJ Open. 2025 Jun 26;15(6):e094788. doi: 10.1136/bmjopen-2024-094788 (PMC12207110; doi:10.1136/bmjopen-2024-094788)
Supplement: online supplemental file 1 [file bmjopen-15-6-s001.pdf]

Brain metastases Questionnaire

This
questionnaire will ask about the
experience of patients with treated brain metastases and their carers. My name is Anna Bangiri and I am a PhD student at the University of Nottingham. My research is focused on assessing the possible long term side effects of radiotherapy treatment in patients with brain metastases. The goal of this research is to identify such side effects with a view to minimising them and improving patient experience where possible. Any answers which you may list in this questionnaire are kept anonymous and confidential. Your replies will not affect the clinical treatment or care you receive.

You do not have to answer every question on the questionnaire. It is helpful if you answer as much as possible if you are comfortable to do so. Please feel free to omit questions that are not relevant to you. If you have any queries about this questionnaire or would like to learn more about my research, please contact me at anna.bangiri1@nottingham.ac.uk

1. What treatment have you(or the person you care/d for) had for your brain metastases?

   Tick all that apply.

   - [ ] Whole Brain radiotherapy
   - [ ] Stereotactic Radiosurgery (including Gamma Knife, Cyberknife, or linac based)
   - [ ] Surgery
   - [ ] Other: ______________________

2. How many separate radiotherapy treatments have you (or the person you care/d for) had for your brain metastases? The focus here is the number of treatments and not the fractions. So for example, you might have had 2 SRS treatments 9 months apart, and Whole Brain Radiotherapy 6 months later. That would be a total of 3 radiotherapy treatments.

   ____

3. What is the origin of your primary cancer?

Mark only one oval.

- ( ) Lung
- ( ) Breast
- ( ) Melanoma
- ( ) Other: ____________________

4. What are the main symptoms that you (or the person you care/d for) have experienced as part of their radiotherapy treatment?

____
____
____
____
____

5. Which symptoms have the largest impact on your daily life?

____

6. Do you feel that your brain function (thinking and/or other activities) has been impacted by the treatment? If yes, please explain in what way.

Tick all that apply.

- [ ] Yes
- [ ] No
- [ ] Other: ____________________

7. If your brain function has been affected, can you tell us how severe is that impact?

Mark only one oval.

	1	2	3	4	5	
Mini	◯	◯	◯	◯	◯	Major long-term impact

8. Has you mental health been impacted upon due to the radiotherapy treatment?

Mark only one oval.

◯ Yes

◯ No

9. If yes, please let us know how (if you are comfortable with this):

____

10. Our team plans to undertake routine cognitive testing for patients with brain metastases. We think that this could help identify possible side effects, or where support may be needed. For this not to be a burden, what interval do you think would be useful and acceptable from a patient's perspective for the testing?

Mark only one oval.

◯ Before treatment and 3-monthly afterwards

◯ Before treatment and 6-monthly afterwards

◯ Before treatment and at 1 year post treatment

◯ Other: ________________________________

11. How long do you think it would be acceptable for the cognitive testing session to last?

Mark only one oval.

- Less than 30 min
- Less than 1 hr

12. Would you prefer the testing to be done in paper form or online via a tablet that would be given to you?

Mark only one oval.

- Online
- Paper form
- Either
- Other: ____________________

13. Where would you like the testing to take place?

Mark only one oval.

- At the hospital, prior to or around the times of other appointments (e.g. CT/MRI scans, follow up appointments)
- At the patient's home
- A mixture
- Other: ____________________

14. Should your Quality of Life (patient and/or carer) measure also be assessed during the testing sessions?

Mark only one oval.

- Yes
- No

15. Please feel free to add any further comments or suggestions you may have here:

16. We kindly thank all participants of this research for taking the time to answer these questions.

This content is neither created nor endorsed by Google.

Google Forms
