# Supplementary material for: Outcomes of patient and public involvement in the development of the Cognitive Decline after Brain Radiosurgery (CoDe B-Rad) study: refining the research question and methodology
Source: BMJ Open. 2025 Jun 26;15(6):e094788. doi: 10.1136/bmjopen-2024-094788 (PMC12207110; doi:10.1136/bmjopen-2024-094788)
Supplement: online supplemental file 2 [file bmjopen-15-6-s002.pdf]

Questionnaire for patients and their carers

This questionnaire is for patients that have had brain radiotherapy and their carers. My name is Anna Bangiri and I am a researcher at the University of Nottingham. I am looking at the long term side effects that radiotherapy treatment has for patients with brain tumours. I am aiming to better focus my research based on patient needs. All the answers to this questionnaire are anonymous and will be kept confidential. You do not have to answer all of the questions on the questionnaire. Feel free to answer only the questions you feel comfortable to do so. If you have any queries about this questionnaire or would like to learn more about my research, please contact me at
anna.bangiri1@nottingham.ac.uk

Untitled section

1. What type of brain cancer does/did you/the person you care for have?

    ____

2. What treatment did you have?

    Tick all that apply.

    - [ ] Surgery
    - [ ] Radiotherapy
    - [ ] Chemotherapy

3. When did you finish your treatment?

    ____

4. If you had radiotherapy, during or after the treatment, what side-effects did you experience? (physical: e.g. unsteadiness, loss of hearing, dizziness, problems walking/holding, loss of strength etc, or mental: e.g. loss of memory, cannot multitask, cannot find the right word, difficulty making decisions etc)

____

____

____

____

____

5. Have any of these side effects improved? If so, which ones?

____

6. Which side effects have not improved?

____

7. How much do they affect your daily life?

Mark only one oval.

	1	2	3	4	5	
not a	◯	◯	◯	◯	◯	daily

8. What is the hardest thing about dealing with the side effects of your treatment?

____

____

____

____

____

9. If you could change something about those side effects, what would it be?

10. What would you like research to focus on with regards to your radiotherapy treatment and its side effects?

11. Is there anything else you would like to add?

This content is neither created nor endorsed by Google.

Google Forms
