# Supplementary material for: Outcomes of patient and public involvement in the development of the Cognitive Decline after Brain Radiosurgery (CoDe B-Rad) study: refining the research question and methodology
Source: BMJ Open. 2025 Jun 26;15(6):e094788. doi: 10.1136/bmjopen-2024-094788 (PMC12207110; doi:10.1136/bmjopen-2024-094788)
Supplement: Supplementary file 3 [file bmjopen-2024-094788.reviewer_comments.pdf]

PEER REVIEW HISTORY

BMJ Open publishes all reviews undertaken for accepted manuscripts. Reviewers are asked to complete a checklist review form and are provided with free text boxes to elaborate on their assessment. These free text comments are reproduced below.

ARTICLE DETAILS

Title (Provisional)

Outcomes of Patient and Public Involvement in the development of the Cognitive Decline after Brain Radiosurgery (CoDe-B-Rad) study: refining the research question and methodology

Authors

Bangiri, Anna; Horobin, Adele; Baker, Julia; Pszczolkowski, Stefan; Thust, Stefanie; Morgan, Paul S

VERSION 1 - REVIEW

**Reviewer**	**1**
**Name**	**Graber, Jerome**
**Affiliation**	**University of Washington, Neurology and Neurosurgery**
**Date**	**13-Dec-2024**
**COI**	**None**

The authors report an attempt to further engage with patients/caregivers in a structured prospective way for input on outcomes and study methods for their concerns in research about cognitive and other effects of stereotactic radiotherapy. They struggled with recruitment for discussion groups and pivoted to small 1 on 1 discussions. This is interesting and worthy of inquiry and further discussion, especially whether the participants they did have might be outliers within this larger population. Despite this detailed input, the researchers receiving this info arrived at the same patient reported outcomes used in the majority of brain tumor trials (MOCA, EORTC QoL forms well validated in various brain tumors). It is worth considering the details of why the researchers arrived at these conclusions and whether the patient/caregiver output actually changed their conceptions or were incorporated.

The study included meningiomas and brain metastases. These are very different clinical disease with very different outcomes. Evaluating the reported symptoms and concerns would be much more informative if they were sorted by these two very different diseases (and potential comparisons could be informative).

Page 11 lists radiotherapy is commonly used for malignant or recurrent meningiomas. Malignant meningiomas are extremely rare, so it is very unlikely that any of these patients had malignant meningiomas. Recurrent benign meningiomas are very common and very commonly treated with radiation.

Page 7: one of the clinical stakeholders was described as "neuro-radiologist". Please clarify if this was a radiation oncologist with direct patient/caregiver contact or a neuroradiologist who typically have no direct interactions with patients and caregivers.

One limitation is that many of these patients are treated with antiepileptic medication and sometimes temporarily with steroids, which are common and significant cofactors in many side effects and symptoms separate from radiation. It does not appear that these were assessed in person or the questionnaire.

**Reviewer**	**2**
**Name**	**Singer, Lauren**
**Affiliation**	**University of Chicago**
**Date**	**20-Feb-2025**
**COI**	**None**

I appreciate the importance of this paper. However, I think some of the value of the discussion is lost in the convoluted methods and overall completion of the trial. I would recommend making the methods and results section more concise so that this can be more easily interpreted and thus practice changing.

VERSION 1 - AUTHOR RESPONSE

Original submission Reviewer: 1

Comments to the Author:
The authors report an attempt to further engage with patients/caregivers in a structured prospective way for input on outcomes and study methods for their concerns in research about cognitive and other effects of stereotactic radiotherapy. They struggled with recruitment for discussion groups and pivoted to small 1 on 1 discussions. This is interesting and worthy of inquiry and further discussion, especially whether the participants they did have might be outliers within this larger population. Despite this detailed input, the researchers receiving this info arrived at the same patient reported

outcomes used in the majority of brain tumor trials (MOCA, EORTC QoL forms well validated in various brain tumors). It is worth considering the details of why the researchers arrived at these conclusions and whether the patient/caregiver output actually changed their conceptions or were incorporated.

Author's response to reviewer No1's first comment: The PPI contributors were not selected by the authors. As explained in the manuscript, national charities were engaged in the recruitment of PPI contributors. The authors had no prior knowledge of who had agreed to participate in the discussion groups but then could not attend. The authors make conclusions on why participants could not attend, in the challenges section of the discussion, based on what was mentioned in the support groups.

The manuscript reads: "Two discussion groups were organised, with approximately 3 weeks' notice given of the date and time but could not go ahead due to late cancellations. One reason could be, as patients explained during the support group sessions, that physical and mental health can vary greatly from one day to the next. The symptoms are inconsistent and unpredictable in their severity. Had there been patient and carer input into developing the initial plans, this issue might have been anticipated and accommodated. This is a limitation of the study, with regards to developing the initial PPI plans."

The authors also mention in the limitations section that due to the low numbers of contributors a diverse range of voices was not achieved. We believe that this covers the reviewer's concerns with regards to the contributors being outliers of the larger population.

With regards to the second part of the comment and how we arrived at the conclusion to use MoCA and EORTC QLQs C30 and BN20, we believe we have demonstrated with our original answer that the tests chosen were selected and incorporated due to the PPI contributions and not preconceived/ decided by the authors.

Original submission Reviewer: 1

The study included meningiomas and brain metastases. These are very different clinical disease with very different outcomes. Evaluating the reported symptoms and concerns would be much more informative if they were sorted by these two very different diseases (and potential comparisons could be informative).

Author's response to reviewer No1's second comment: Thank you for your helpful comment. Reviewer No 1 kindly points out that meningiomas and brain metastases are two different conditions. Indeed, reviewer No1 is correct. In our original response, we recognise the difference of the two conditions by separating the population in two different cohorts. There has been no mention of the two cohorts in the manuscript, as the manuscript aims to show the PPI aspect in the development of the protocol but does not go into the details of the study protocol itself.

In this revision, we have added a brief mention of the two cohorts in the Results section-subsection Defining the patient population, that reads: "To distinguish between long- and short-term side effects and different life expectancies of patients, two cohorts will be defined.

A retrospective cohort, consisting solely of meningioma patients, and a prospective cohort, consisting of meningioma and brain metastases patients."

**Please further revise the article title to better clarify the purpose and scope of the present manuscript. Eg, 'Refining the research question and methodology of a study to identify potential side-effects of stereotactic radiosurgery: outcomes of patient and public involvement in the development of the Cognitive Decline after Brain Radiosurgery Study (CoDe-B-Rad)' (or similar, please amend as needed).*

Author's response: Thank you for the kind suggestion, this has now been amended to read: "Outcomes of Patient and Public Involvement in the development of the Cognitive Decline after Brain Radiosurgery (CoDe-B-Rad) study; refining the research question and methodology."

**Please update the abstract to (i) include a 'Design' section after the 'Objectives' section; (ii) include more details in the 'Setting' section (eg, country, timing of the conduct of the work (the current status of the CoDe-B-Rad study would also be appropriate to mention here, as well as in the main text, for the sake of clarity); and (iii) update the 'Trial registration' section to clarify if this is referring to the registration of the CoDe-B-Rad study, rather than the present PPI work specifically.*

Authors' response: Thank you for your comments please see our replies for each item below: (i)A design section has been added and it reads" Patient and Public Involvement".

(ii) This has now been amended to read: "PPI conducted online with people based in the United Kingdom. CoDe-B-Rad study running in regional NHS tertiary care, in the UK, and is currently nearing recruitment completion."

(iii) This has now been amended to read: "Clinicaltrial.gov NCT06466720. This is referring to the registration of the CoDe-B-Rad study and not the present PPI work, which does not require registering in the United Kingdom."

*Please change the heading 'Article Summary' to 'Strengths and limitations of this study' and please remove the 'Strengths' and 'Limitations' subheadings (it should be clear from their content which are which).

Author's response: Apologies for missing this our previously, this has now been amended.

**Can you clarify why informed consent was not obtained from the participants?*

Author's response: Please see section titled Ethics approval at the end of the manuscript which states the following: "Patient and Public involvement activities are not considered research nor collection of study data in the UK as per the National Institute of Health Research (NIHR) and the Health Research Authority[49][50]. Consequently, ethical approval is not required for the PPI activities. Informed consent was not obtained from any of the contributors. Subsequently, ethical approval was obtained for the CoDe-B-Rad study (IRAS 340408, London Stanmore REC Reference 24/PR/0426)."

Should you require any more clarification please do not hesitate to contact us.
**

Reviewer: 2
Dr. Lauren Singer, University of Chicago
Comments to the Author:
I would recommend editing the results section of the abstract. Some of the wording is confusing, and I think this is due to the changes in tense (present/past).

Author's response: Thank you for your kind comment. The results section of the abstract has been amended with all sentences written in the past tense.

Additionally, slightly niche, but the paper states that all patients with brain mets are not allowed to drive (page 16). This is not correct and only applies to patients with significantly poor KPS or who have had generalized convulsions. Would qualify that "in some instances"

Author's response: Thank you for your comment. In the UK, the government states that patients with brain metastases are only allowed to drive if they have asymptomatic lesions that have been picked up on routine imaging. If they have had radiotherapy as treatment they are not allowed to drive (Neurological disorders: assessing fitness to drive - GOV.UK). The statement was made to put in context the decision to offer testing at home to minimise the burden to participants.

We have amended the text to read: "In the UK, patients who have received SRS are not allowed to drive".

*** ***

VERSION 2 - REVIEW

**Reviewer**	**2**
**Name**	**Singer, Lauren**
**Affiliation**	**University of Chicago**
**Date**	**11-Apr-2025**
**COI**	

I would recommend editing the results section of the abstract. Some of the wording is confusing, and I think this is due to the changes in tense (present/past).

Additionally, slightly niche, but the paper states that all patients with brain mets are not allowed to drive (page 16). This is not correct and only applies to patients with significantly poor KPS or who have had generalized convulsions. Would qualify that "in some instances"

VERSION 2 - AUTHOR RESPONSE

Reviewer: 2
Dr. Lauren Singer, University of Chicago
Comments to the Author:
I would recommend editing the results section of the abstract. Some of the wording is confusing, and I think this is due to the changes in tense (present/past).

Author's response: Thank you for your kind comment. The results section of the abstract has been amended with all sentences written in the past tense.

Additionally, slightly niche, but the paper states that all patients with brain mets are not allowed to drive (page 16). This is not correct and only applies to patients with significantly poor KPS or who have had generalized convulsions. Would qualify that "in some instances"

Author's response: Thank you for your comment. In the UK, the government states that patients with brain metastases are only allowed to drive if they have asymptomatic lesions that have been picked up on routine imaging. If they have had radiotherapy as treatment they are not allowed to drive (Neurological disorders: assessing fitness to drive - GOV.UK). The statement was made to put in context the decision to offer testing at home to minimise the burden to participants.

We have amended the text to read: "In the UK, patients who have received SRS are not allowed to drive".
